# The Effect of Venipuncture Site on Hematology of Bats: Implications for Comparative Analyses

**DOI:** 10.1093/icb/icaf026

**Published:** 2025-05-19

**Authors:** Alicia Roistacher, Bret Demory, Daniel J Becker

**Affiliations:** School of Biological Sciences, University of Oklahoma, 730 Van Vleet Oval, Norman, OK 73019, USA; School of Biological Sciences, University of Oklahoma, 730 Van Vleet Oval, Norman, OK 73019, USA; School of Biological Sciences, University of Oklahoma, 730 Van Vleet Oval, Norman, OK 73019, USA

## Abstract

Wildlife health comparisons within and across populations and species are essential for population assessment and surveillance of emerging infectious diseases. Due to low costs and high informational yield, hematology is commonly used in the fields of ecoimmunology and disease ecology, yet consistency and proper reporting of methods within these fields are lacking. Previous investigations on various wildlife taxa have revealed noteworthy impacts of the vein used for blood collection on hematology measures. However, the impacts of venipuncture site on bats, a taxon of increasing interest in ecoimmunology and disease ecology, have not yet been tested. Here, we use a long-term study system in western Oklahoma to test the effect of venipuncture site on hematology parameters of the Mexican free-tailed bat (*Tadarida brasiliensis*) and cave myotis (*Myotis velifer*), two abundant and representative bat species from the families Molossidae and Vespertilionidae. Between September 2023 and October 2024, we collected paired peripheral blood from both the propatagial and intrafemoral veins in 25 individuals per species. We then quantified total red and white blood cells, reticulocyte counts, and leukocyte differentials and used generalized linear mixed models to compare parameters among venipuncture sites within and between bat species. Overall, venipuncture site had no effect on any hematology parameters; however, we revealed small differences in neutrophil and lymphocyte proportions between veins among the species. By contrast, we detected significant species-level differences in most cell measurements, which we propose could be explained by life-history strategy and phylogenetic differences. We encourage continued testing of additional venipuncture sites, and of the same venipuncture sites on different species, on hematology and other health metrics used in ecoimmunology and disease ecology. Lastly, we emphasize the importance of thorough method reporting in publications to enable transparent comparisons and accounting for even small sampling-based artifacts. All future efforts are especially important for bats to improve conservation monitoring, ecosystem services estimations, and their association with emerging infectious diseases.

## Introduction

Hematology is widely used in the fields of ecoimmunology and disease ecology owing to its use of non-lethal, low-cost, and small volume blood samples (Maceda-Veiga et al. 2015; Kloskowski et al. 2017). Peripheral blood from vertebrates reflects whole-organism function and can be subject to many assays, including but not limited to differential white blood cell (WBC) and red blood cell (RBC) counts, blood chemistry panels, platelet counts, comet assays, and parasite screening (Maceda-Veiga et al. 2015; Gupta and Nigar 2020; Dziki-Michalska et al. 2024). The data obtained from these assays can provide insights into innate and adaptive immune function, intrinsic and extrinsic stressors, and parasite infections (Kloskowski et al. 2017; Gupta and Nigar 2020). Blood smears are the most widely used sample when studying wildlife health due to their low costs and small blood requirement relative to their high information yield (Maceda-Veiga et al. 2015). For example, ratios of neutrophils to lymphocytes (NL ratios) are commonly used to assess stress and immunity (Davis et al. 2008; DeAnglis et al. 2024; Tovstukha et al. 2024), while RBCs, reticulocytes (RETs), and WBCs are often used to diagnose disease (Pouletty 2010) as well as detect blood parasites (Schinnerl et al. 2011; Gupta and Nigar 2020; Alharbi et al. 2022), inflammation, and tissue damage (Maceda-Veiga et al. 2015).

The vein used to collect peripheral blood (i.e., venipuncture site) can have a significant effect on hematological and biochemical parameters in diverse wildlife taxa, including but not limited to turtles (Rodríguez-Almonacid et al. 2022), tortoises (Gottdenker and Jacobson 1995; López-Olvera et al. 2003; Eshar et al. 2016; Neiffer et al. 2021), sharks (Mylniczenko et al. 2006), deer (Comazzi et al. 2023), and birds (Kern and De Graw 1978; Sheldon et al. 2008). For example, in the Colombian slider (*Trachemys callirostris*), hematocrit, haemoglobin, and RBC count significantly varied between three vein types (Rodríguez-Almonacid et al. 2022). Furthermore, other hematology studies not directly testing for venipuncture have hypothesized that their observed cell differences could be explained by differing venipuncture sites (Gregory et al. 2022; Crooks et al. 2023). Owing to different protocols and logistical constraints, sample collection and storage methods are not standardized across and within research groups or across seasons, populations, and/or species within groups (Maceda-Veiga et al. 2015; Rodríguez-Almonacid et al. 2022). Testing for the potential effects of venipuncture site on hematology across wildlife taxa more generally is important to account for such sampling artifacts in existing and future analyses (Neiffer et al. 2021). Improving upon method standardization, increased efforts in baseline data collection, and reporting of venipuncture type used will enhance the fields of ecoimmunology and disease ecology and improve inference of comparative analyses.

Bats are increasingly studied in ecoimmunology and disease ecology due to their ecological diversity and association with emerging infectious diseases, especially as some species seem to tolerate virulent viruses with little-to-no pathology (Irving et al. 2021; Becker et al. 2025b). Hematological methods have been commonly used to assay the bat cellular immune system and response to both intrinsic and extrinsic factors, given the lack of bat-specific immunological reagents, remote field sites and limited cold chain capacity, and the small blood volumes that can be safely collected from many bats (Schneeberger et al. 2013; Ruoss et al. 2019). Hematology has been used to characterize cell types for an increasing number of bat species while documenting the range of variation observed in the morphology of each cell type in bat blood (Schinnerl et al. 2011; Uzenbaeva et al. 2019; Hansen et al. 2022). Cellular immune profiles significantly vary across bat species due to their diverse ecological characteristics, evolutionary histories, and geographical distributions (Schneeberger et al. 2013; Cornelius Ruhs et al. 2021; Becker et al. 2025b). For example, prior work has revealed annual trends in total and differential WBC counts that varied by bat species within the Neotropics (DeAnglis et al. 2024). Additionally, differential WBC counts in Neotropical bats vary by diet (Schneeberger et al. 2013; DeAnglis et al. 2024) but show mixed relationships with body mass (Schneeberger et al. 2013; Cornelius Ruhs et al. 2021). However, despite increasing research efforts on bat cellular immunity, hematology characterization across the bat phylogeny remains limited (Cornelius Ruhs et al. 2021).

**Fig. 1:**
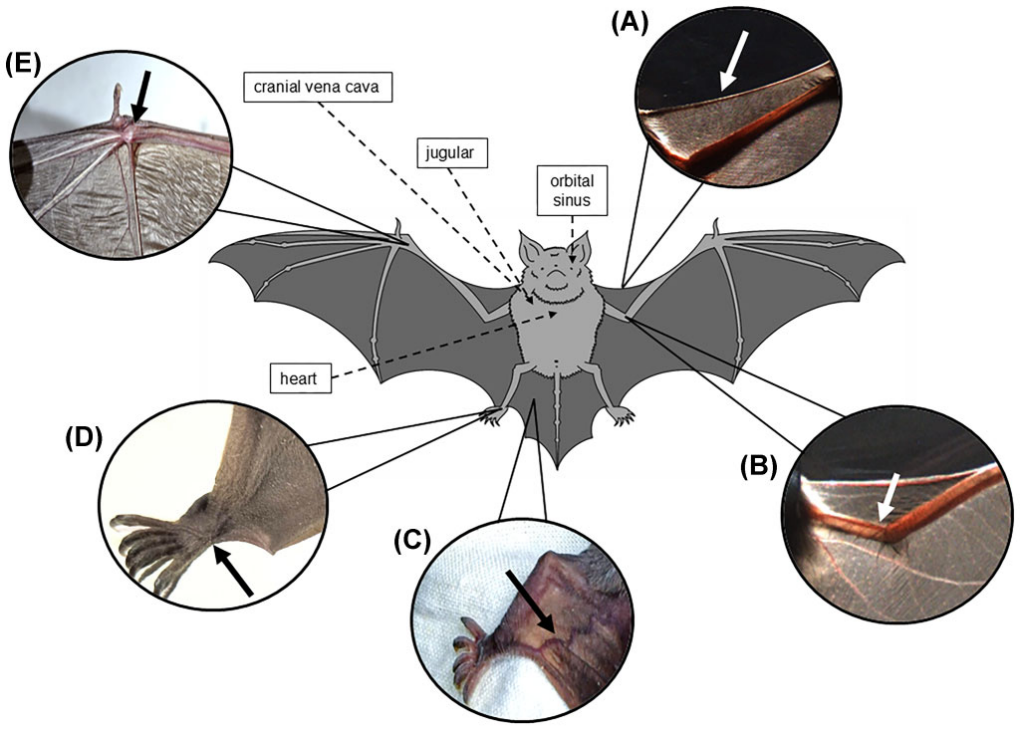
Depiction of all venipuncture sites in bats. The externally visible veins are (A) propatagial (*photo: Ralph Simon)*, (B) brachial (*photo: Ralph Simon*), (C) intrafemoral (*photo: Jon Alonzo*), (D) lower intrafemoral, and (E) upper brachial. The other veins that require lethal blood collection are shown with dashed lines. Here, we focus on hematological differences collected from the propatagial (A) and intrafemoral (C) veins.

Due to personal preference as well as bat body mass and morphology, different veins are typically used to collect peripheral blood (Fig. 1). Conventionally, bats are non-lethally bled from either the propatagial (cephalic) or intrafemoral (saphenous) vein, but they are also lethally bled from cranial vena cava, heart, jugular, and orbital sinus veins (Eshar and Weinberg 2010). This between- and even within-study variation could introduce further noise and biases for comparative analyses and determination of baseline data for newly discovered or underrepresented species. Further, to our knowledge, the effect of venipuncture site on any health metric in bats has not been investigated.

In this study, we test the effect of the two most commonly used veins for bat blood collection, the propatagial and intrafemoral venipuncture sites, on hematology measures of two sympatric bat species, the Mexican free-tailed bat (*Tadarida brasiliensis*) and cave myotis (*Myotis velifer*). To our knowledge, this represents the first assessment of paired hematology values collected from two venipuncture sites in any bat species. We also contribute baseline hematology measures of these two bat species during critical life-history events for ongoing intra- and inter-specific studies.

## Methods

### Study species and sites

We focused our study on two seasonally sympatric bat species in western Oklahoma as part of a long-term study of bat immunity and infection (Becker et al. 2025a). The Mexican free-tailed bat (*T. brasiliensis*), a member of the Molossidae family, migrates from their wintering grounds in central and southern Mexico to the southwestern United States and northern Mexico to give birth and raise their offspring (Villa and Lendell Cockrum 1962; Wiederholt et al. 2015). In addition to long-distance migration, *T. brasiliensis* form the largest congregations of any mammal, containing up to tens of millions of individuals in per colony (Ammerman et al. 2012; Iskali and Zhang 2015; McCracken et al. 2018; Danielson et al. 2022). By contrast, the cave myotis (*M. velifer*) is part of the Verspertilionidae family and inhabits arid temperate and semi-tropical regions from central Oklahoma to southern California in the United States and into southern Mexico (Krutzsch 2009). *Myotis velifer* enter torpor and hibernate during winter (Fitch et al. 1981; Krutzsch 2009), although they have been observed undergoing short-distance or altitudinal migrations to their hibernacula (Ayala-Berdon and Solis-Cardenas 2017). Compared to *T. brasiliensis, M. velifer* form smaller colonies of up to 10,000 individuals per group (Humphrey and Oli 2015), but these are large colonies for the genus *Myotis* (Santana et al. 2011; Cheng et al. 2021; Burrell and Bergeson 2022). Both species are insectivores, contributing to significant reduction of annual agricultural costs of pesticides (Wiederholt et al. 2015; Danielson et al. 2022). Additionally, *M.veilfer* and *T. brasiliensis* are often found co-roosting in caves and anthropogenic dwellings (Betke et al. 2024), share similar reproductive characteristics, and form maternity colonies during summer months (Fitch et al. 1981; Wilkins 1989).

Bats were captured at the Selman Bat Cave and Alabaster Caverns State Park in Woodward County, Oklahoma. Woodward County sits within the Cimarron Gypsum Hills karst area of Oklahoma (William and Samanie 2010). Both caves have exposed gypsum, are overlain with shale layers, surrounded by sage bush, cattle pasture, and high rolling plains separated by eroded canyons (Caire et al. 1984; William and Samanie 2010; Bryant 2013). These sites were chosen based on the large populations of *T. brasiliensis* and *M. velifer* inhabiting the caves. The Selman Bat Cave houses an estimated 50,000 *T. brasiliensis* each year over their summer occupancy (Betke et al. 2008), and the cave system we used at Alabaster Caverns State Park houses multiple bat species, including both *T. brasiliensis* and *M. velifer* from spring into early autumn (Caire et al. 1984). Caves in Woodward County have been reported as hibernacula for *M. velifer* (Caire and Loucks 2010; Humphrey and Oli 2015). There is a lack of migration reports for this species in northwest Oklahoma; thus, we assume *M. velifer* strictly hibernates here.

We sampled bats between September 2023 and October 2024. Bats were captured with hand nets at the Selman Bat Cave and with hand nets and mist-nets at the cave at the Alabaster Caverns State Park. Both sexes (*M. velifer*: 36% female, 64% male; *T. brasiliensis*: 40% female, 60% male) and all reproductive groups (*M. velifer*: 76% non-reproductive, 24% reproductive; *T. brasiliensis*: 96% non-reproductive, 4% reproductive) were included in this study; all bats were adults. We determined our sample size (*n* = 25 paired samples per species; *n* = 50 paired samples total) using a two-sided, two-sample power analysis, assuming 92% power.

### Sample collection

We collected paired blood from both the propatagial and intrafemoral veins per each individual bat (Fig. 1). Before bleeding, veins were sterilized with 70% isopropyl and lanced with a sterile 26G needle, then blood was directly collected in a heparinized capillary tube. We used approximately 2 μL blood to make a thin blood smear on a sterile microscope slide, and the remaining blood was immediately transferred to a sterile 0.2 mL PCR tube to be used for total RBC, WBC, and RET counts. Because consecutive bleeds and relatively longer lags between animal capture and bleeding (e.g., bats bled later in the night) can induce physiological stress and affect cellular immunity (Davis 2005; Davis et al. 2008), we recorded bleeding order from the propatagial and intrafemoral veins as well as the time between bat capture and blood collection. We aimed to collect an even ratio of bleeding order to reduce potential bias, and bats were on average held for 3.7 h prior to blood collection.

The bat research community has closely followed the universal rule for blood collection across vertebrates: do not collect more than 1% of the animal’s body weight in blood within a 24-h period (Wolfensohn and Lloyd 2013; Sikes 2016). Handling and care of bats were followed as described in Guidelines of the American Society of Mammalogists for the use of wild mammals in research (Sikes 2016). All work was approved under University of Oklahoma Institutional Animal Care and Use Committee (IACUC) protocol 2022–0198 and scientific collection permit 10567389 from the Oklahoma Department of Wildlife Conservation. Proper personal protective equipment, KN95 masks, and leather and nitrile gloves were worn while handling and collecting samples to limit parasite spread to or from bats (Olival et al. 2020).

### Hematology measurements

To increase the accuracy of total RBC, WBC, and RET counts, we coupled blood smear analyses with Neubauer chamber counts (Fisher Scientific; depth 0.1 mm, area 0.0025 mm^2^). Blood was diluted 1:100 in a NaCl/EosinY solution for RBC counts and 1:1200 in a NaCl/Crystal Violet solution for WBC and RET counts (Table S1; Table S2). Sodium chloride and dye stock solutions were made no longer than 1 day before use, and dye stocks were stored in dark vessels. For consecutive nights of sampling in the field, stock solutions were stored in 4°C but not reused more than once. Within 1–2 h of blood collection, each sample was stained and read for WBC, RBC, and RET total counts. WBC counts were calculated by averaging the number of cells observed in the four large 1 × 1-mm squares on either corner of the chamber; this equaled one read (Hansen 2000). RBC and RET counts were calculated by averaging the number of cells observed in the five smaller 0.2 × 0.2-mm squares within the large middle square of the chamber; each five square average count equaled one read (Hansen 2000). Each cell measurement was read under 400X magnification with a binocular microscope (Euromex EBS1152EPLI). We used the following equations to calculate cells per microliter (Hansen 2000):


\begin{eqnarray*}
{\mathrm{RBCs}}/\mu {\mathrm{L}} &=& ( {\mathrm{median}}\,{\mathrm{cell}}\,{\mathrm{count}}*5*{\mathrm{dilution}}\,{\mathrm{factor}}\\
&& * 5 \times {10}^{4} )/ 1 \times {10^3}
\end{eqnarray*}

\begin{eqnarray*}
{\mathrm{WBCs}}\,{\mathrm{or}}\,{\mathrm{RETs}}/\mu {\mathrm{L}} &=& ( {\mathrm{median}}\,{\mathrm{cell}}\,{\mathrm{count}}*4\\
&&*{\mathrm{dilution}}\,{\mathrm{factor}}*1 \times {10}^{4})/1 \times {10^3}.
\end{eqnarray*}

Blood smears were air dried and stored in sealed boxes with desiccant packs until they were fixed with methanol and stained with Wright–Giemsa (Quick III, Astral Diagnostics). To obtain WBC differentials, we identified up to 100 leukocytes, only counting cells in the monolayer of each blood smear under 1000X magnification (oil immersion) in a systematic grid pattern from head to tail in strips, ensuring not to overlap when moving into a new viewing strip (Bain et al. 2006). Blood surface area of blood smears was not standardized, and some samples, despite having a large blood surface area, did not have 100 WBCs to identify. For downstream analyses, we categorized each blood smear by its surface area (small or large). All Neubauer chamber and blood smear measures were determined by one reader.

### Statistical analysis

All analyses were performed in R software (R Core Team 2023). To first assess consistency of each cell measure between venipuncture sites, we calculated intraclass correlation coefficients (ICCs) within each bat species using the *iccCounts* package (Carrasco 2022). We classified levels of consistency as previously described: high (ICC ≥ 0.9), good (0.75 ≤ ICC ≤ 0.9), moderate (0.5 ≤ ICC ≤ 0.75), and poor (ICC < 0.5) (Haghayegh et al. 2020). We next used generalized linear mixed models (GLMMs) fit using the *lme4* package (Zuur 2009) to test fixed effects of venipuncture site, bat species, and their interaction on all cell counts. All GLMMs included a random intercept for bat identification number and a random slope of venipuncture site. For total RBC, WBC, and RET counts, we used the median value across each read per sample; RBC and RET counts were modeled as negative binomial responses, while WBC counts were analyzed with a Poisson response, based on relationships between the cell count means and variance. WBC differential counts were modeled as binomial responses, considering the counts of each cell relative to that of all other cells identified; this approach allowed us to account for variation in total number of cells identified. We conducted post-hoc analyses and obtained marginal means from each GLMM using the *emmeans* package (Lenth et al. 2018). We then calculated the contrast percentage of the marginal mean values between veins within each species.

Though aiming to obtain an even ratio of vein bleeding order within our sample size goal (*n* = 30 paired samples per species), our final ratio for both species was slightly uneven: 10 intrafemoral and 40 propatagial samples for *M. velifer*, 20 intrafemoral and 30 propatagial samples for *T. brasiliensis*. To assess the potential bias of bleeding order ratio, we excluded data from the first sampling trip (September 2023) to obtain a more even ratio: 10 intrafemoral and 30 propatagial samples for *M. velifer*, 20 intrafemoral and 20 propatagial samples for *T. brasiliensis*. Likewise, we ran additional sensitivity analyses that independently included blood smear size, vein bleeding order, time between capture and blood collection, and bat sex as precision covariates; all covariates could not be included in the same GLMM due to sample size.

## Results

**Fig. 2:**
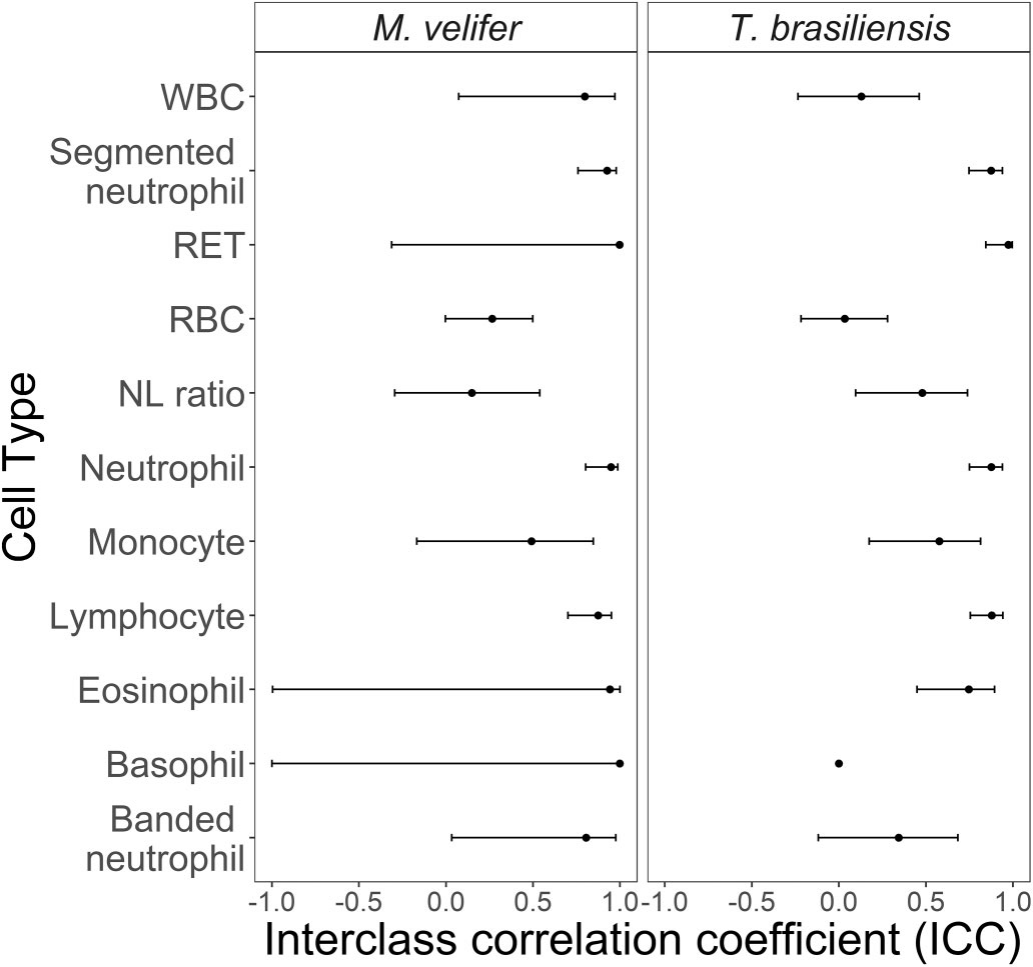
Repeatability estimates (ICCs) and 95% confidence intervals for each cell type. Where confidence intervals are not present, values were exponentially negative.

When assessing concordance in cell counts between propatagial and intrafemoral veins for *M. velifer*, consistency was high for RETs, basophils, eosinophils, segmented neutrophils, and total neutrophils; good for WBCs, banded neutrophils, and lymphocytes; moderate for monocytes; and poor for RBCs and NL ratios (Fig. 2). Similarly, for *T. brasiliensis*, RETs were highly consistent, while eosinophils, segmented neutrophils, lymphocytes, and total neutrophils had only good consistency. Monocytes again were moderately consistent, and RBCs again had poor consistency. In contrast to *M. velifer*, WBCs, basophils, and banded neutrophils had poor consistency (Fig. 2).

**Fig. 3:**
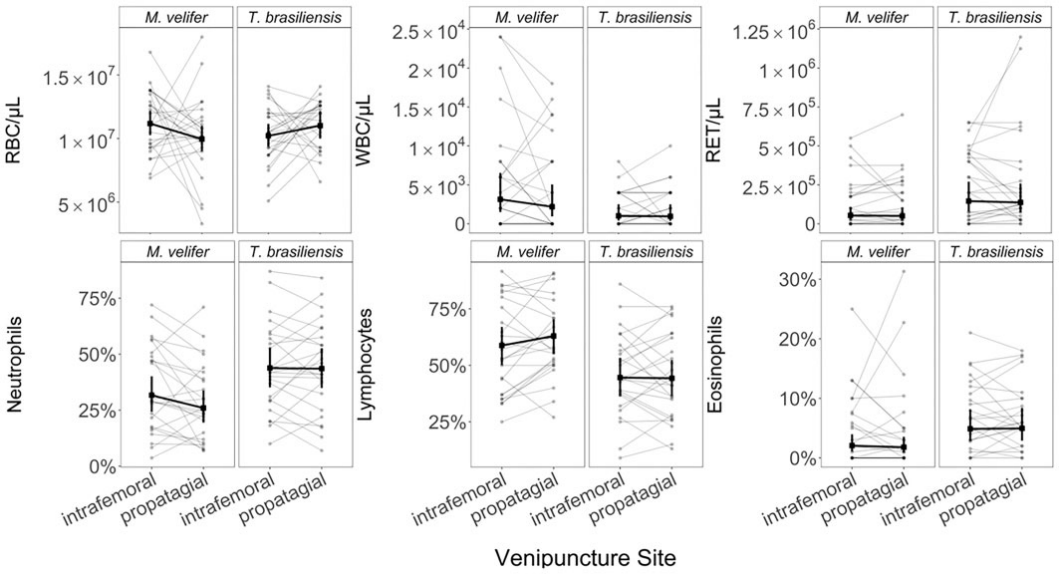
Hematology values as a function of venipuncture site, stratified by bat species. Paired vein data for an individual bat are shown through line segments. Bold coloring indicates the predicted means and 95% confidence intervals for each vein per species from our GLMMs. Summary statistics are provided in Table 1 for effects of venipuncture site, bat species, and their interaction.

Predicted means and confidence intervals of each cell measurement from our GLMMs are shown in Fig. 2 and Fig. S1. Across all our hematology values, our GLMMs found no significant effects of venipuncture site within or across our two bat species (Table 1 and Table S3). However, we did see species-level differences in WBC counts, RET counts, neutrophils, lymphocytes, NL ratios, segmented neutrophils, eosinophils, and basophils (Table 1 and Table S3). Results were consistent when analyzing only the subset of our data with a more even ratio of vein bleeding order (Table S4). Similarly, results were unaffected when blood smear size, vein bleeding order, time between capture and blood collection, or sex were independently accounted for in our GLMMs (Figs. S2 and S3; Tables S5–S7). Sex and reproductive status are dependent in these data; therefore, only sex was included in analyses (Pearson’s chi-squared test: χ^2^ = 1.94, df = 1, *P* = 0.16).

**Table 1:** Summary of GLMM results in Fig. 3, using type II ANOVA tests

Cell measure	Variable	χ^2^	df	*P*
RBC	Vein	0.1402	1	0.70811
	Species	0.0112	1	0.91569
	Vein:Species	3.3425	1	0.06751
WBC	Vein	0.4268	1	0.51355
	Species	**4.8307**	**1**	0.02796*
	Vein:Species	0.3482	1	0.55514
RET	Vein	0.1196	1	0.72944
	Species	**5.0185**	**1**	0.02508*
	Vein:Species	0.0006	1	0.98037
Neutrophils	Vein	1.8590	1	0.172739
	Species	**7.0288**	**1**	0.008021**
	Vein:Species	1.8184	1	0.177506
Lymphocytes	Vein	0.8175	1	0.36591
	Species	**9.4342**	**1**	0.00213**
	Vein:Species	1.2047	1	0.27238
Eosinophils	Vein	0.0106	1	0.91813
	Species	**5.6145**	**1**	0.01781*
	Vein:Species	0.2450	1	0.62059

Using marginal means from our GLMMs, we observed the largest inter-vein difference for the NL ratio in *T. brasiliensis*, where NL ratios from the propotagial vein were 67% higher than those from the intrafemoral vein (Fig. S1). For all other cell measurements, our GLMMs identified very small inter-vein differences: RBCs (2.7%); RETs, neutrophils, and lymphocytes (0.3%); monocytes and segmented neutrophils (0.2%); banded neutrophils (0.11%); eosinophils (0.08%); basophils (0.05%); and WBCs (0.02%). We observed similarly small inter-vein differences in *M. velifer*: RBCs (4.1%), monocytes (0.5%), banded neutrophils (0.42%), eosinophils (0.3%), WBCs (0.23%), basophils (0.2%), and RETs (0.1%). In comparison to *T. brasiliensis*, however, *M. velifer* had a higher percent contrast for mean cell counts between veins for neutrophils (5.7%), segmented neutrophils (4.7%), and lymphocytes (4%), but this species had a lower percent contrast for NL ratios (1.6%).

## Discussion

Standardized sampling protocols and proper methods reporting are important to facilitate robust comparisons in the fields of ecoimmunology and disease ecology. Our findings emphasize the relevance of these points for studies of wild bats, due to the high frequency of hematological techniques used to infer health status and infection prevalence in research on the chiroptera and emerging infectious diseases (Kovalchuk et al. 2017; Villalba-Alemán et al. 2020; DeAnglis et al. 2024). Here, we tested the effect of two of the most common venipuncture sites in bats, the propatagial and intrafemoral veins, on multiple hematology measures of two tractable temperate bat systems in the Americas. We found that most cellular measures were highly repeatable, and all our GLMMs found no effect of venipuncture site on any of our hematology parameters. By contrast, we did find significant differences between bat species for many cell measures. We chose our two sample species (*T. brasiliensis* and *M. velifer*) not only based on economical and practical feasibility, as they are involved in long-term projects on bat immunity and infection (Becker et al. 2025a), but also to capture variability within two of the largest and globally distributed chiropteran families, the Molossidae (*T. brasiliensis*) and Vespertilionidae (*M. velifer*). As aforementioned, *T. brasiliensis* and *M. velifer* vary in multiple ecological traits, such as their life-history strategy (i.e., long-distance migration and lack of hibernation versus resident or short-distance migratory status and hibernation). The significant differences detected among multiple cell measures between these two species could thus be attributed to their unique wintering strategies (Auteri 2022). Furthermore, we are uncertain what could explain the noteworthy difference in the estimated means of the neutrophil and lymphocyte counts by venipuncture site between *T. brasiliensis* and *M. velifer*. Given similar capture and sample collection procedures among these two species, these results could be another cause of taxonomic family or life-history strategy differences. Future comparisons of cellular immunity in more species representatives within the Molossidae and Vespertilionidae, as well as targeted seasonal sampling, could address these observed differences.

Based on the ubiquitous lack of venipuncture effects across hematology measurements, our findings suggest little to no differences in cellular pathways or composition throughout the body of *T. brasiliensis* or *M. velifer*. Previous studies in species other than bats suggest that lymph hemodilution, lymph contamination (Neiffer et al. 2021; Rodríguez-Almonacid et al. 2022; Crooks et al. 2023), hemolysis, and sera dilution (Comazzi et al. 2023) could explain different hematology values at each vein site due to their proximity to lymphatics. Other work has suggested that microanatomical obstacles in the circulatory system could explain observed hematological differences by venipuncture site (Mylniczenko et al. 2006), and our null findings imply that such effects may not be present among these sympatric but phylogenetically distinct species

When conducting our comparisons across venipuncture sites within and across bat species, we aimed to eliminate as many biases as possible, including statistically powerful and equal sample-size groups for each species, ensuring identical methods were carried out for each blood draw, and having only one reader for all hematology metrics. We also considered bleeding order of the veins, time between capture and bleeding, and differences in blood smear size, with results consistent after statistically adjusting for these variables. However, we acknowledge some limitations still remain in these data, such as not having an equal bleeding order ratio in the final dataset, sampling occurring during different months of the year, variation in handler and bleeder, and variability in the time between blood collection and Neubauer chamber readings.

Given our focus on two bat species within two families for two commonly used veins in blood collection, we encourage testing of venipuncture sites on other veins used in bat surveys, testing between age, reproductive status, and sex groups, and expanding these tests to other bat species (and families) to further support the non-significant effects observed here. We also encourage testing effects of venipuncture site on health metrics other than hematology that are increasingly used in bat immunology, such as gene expression and protein abundance data afforded through transcriptomics and proteomics (Arnold et al. 2018; Becker and Banerjee 2023). Future studies testing venipuncture sites in bats should also consider the limitations we note above. Any significant findings of future studies could be used to determine a correction factor between vein types, which could then be applied more generally to ensure comparisons of health metrics in bats are robust and appropriately assessed.

Lastly, our results should provide some reassurance for the fields of ecoimmunology and disease ecology, as our findings suggest negligible effects of blood collection from either the propatagial or intrafemoral vein on typical hematology measurements. Because of this, we believe there should be negligible miscalculation or interpretation of results from previous hematology studies with molossid or vesper bat species that collected peripheral blood from a combination of the propatagial and intrafemoral veins or for comparative analyses where different studies use one or the other vein for blood collection. These findings are also valuable for future similar intra- and inter-species comparative analyses, as vein type would not be a necessary precision covariate (Becker et al. 2025b). However, we suggest that vein type should still be consistently reported in publications, and it could still be valuable to establish baseline parameter ranges by each vein site (Neiffer et al. 2021). For species of conservation concern, researchers could use our findings to motivate switching vein types for blood collection during sample collection to obtain the minimum blood requirements for assays to inform health status (Ohmer et al. 2021).

## Supplementary Material

icaf026_Supplemental_Files

## Data Availability

The data that support the findings of this study are openly available in Dryad with the identifier 10.5061/dryad.tqjq2bw9q.

## Notes

From the symposium “Applied Ecoimmunology in Wildlife Health and Conservation” presented at the annual meeting of the Society for Integrative and Comparative Biology, January 3-7, 2025 at Atlanta, Georgia.
