# Supplementary material for: The Effect of Venipuncture Site on Hematology of Bats: Implications for Comparative Analyses
Source: Integr Comp Biol. 2025 May 19;65(6):1843–52. doi: 10.1093/icb/icaf026 (PMC12690471; doi:10.1093/icb/icaf026)
Supplement: icaf026_Supplemental_Files [file icaf026_supplemental_files.zip › icb-2025-0043-File009.docx]

**The effect of venipuncture site on hematology of bats: implications for comparative analyses: Supplemental Material**

Alicia Roistacher, Bret Demory, and Daniel J. Becker

|  |
| --- |
| **Figure S1:** Hematology values as a function of venipuncture site, stratified by bat species. Paired vein data for an individual bat are shown through line segments. Bold coloring indicates the predicted means and 95% confidence intervals for each vein per species from our GLMMs. GLMM results are provided in Table S3 for effects of venipuncture site, bat species, and their interaction. |

|  |
| --- |
| **Figure S2:** Hematology values as a function of venipuncture site, stratified by bat species. Paired vein data for an individual bat are shown through line segments. Bold coloring indicates the predicted means and 95% confidence intervals for each vein per species from our GLMMs. Data are the same as in Figure 2. Nodes are colored by sex, where females are green and males are yellow. |

|  |
| --- |
| **Figure S3:** Hematology values as a function of venipuncture site, stratified by bat species. Paired vein data for an individual bat are shown through line segments. Bold coloring indicates the predicted means and 95% confidence intervals for each vein per species from our GLMMs. Data are the same as in Figure S1. Nodes are colored by sex, where females are green and males are yellow. |

| **Table S1**: Formulas for Neuber chamber dye solutions. All volumes provided in μL units. Blood/dye solutions were mixed in 2mL vessels. Blood was added within 5 minutes of counting, the vessel was gently inverted to sufficiently mix, and then given ~2 minutes to allow cells to absorb the stain. Blood/dye solutions are loaded into the Nauber chamber then left for another ~1-2 minutes to allow the cells to evenly disperse in the chamber before counting. | | | | |
| --- | --- | --- | --- | --- |

| **Table S2:** Formulas for 10mL stock solutions of Neuber chamber dyes. Units per reagent is provided within each cell. | | | | | | |
| --- | --- | --- | --- | --- | --- | --- |

| **Table S3**: Summary of GLMM results for cell measures included in Figure S1, using type II ANOVA tests. | | | | |
| --- | --- | --- | --- | --- |

| **Table S4:** Summary of GLMM results for cell measures fit to the subset of data with a more even ratio of vein bleeding order, using type II ANOVA tests. | | | | |
| --- | --- | --- | --- | --- |

| **Table S5:** Summary of GLMMs that include blood smear size, using type II ANOVA tests. | | | | |
| --- | --- | --- | --- | --- |

| **Table S6:** Summary of GLMMs that include vein bleeding order, using type II ANOVA tests. | | | | |
| --- | --- | --- | --- | --- |

| **Table S7:** Summary of GLMMs that include time between capture and blood collection (holding time), using type II ANOVA tests. | | | | |
| --- | --- | --- | --- | --- |

| **Table S8:** Summary of GLMMs that include bat sex, using type II ANOVA tests. | | | | |
| --- | --- | --- | --- | --- |
