# Supplementary material for: The Effect of Venipuncture Site on Hematology of Bats: Implications for Comparative Analyses
Source: Integr Comp Biol. 2025 May 19;65(6):1843–52. doi: 10.1093/icb/icaf026 (PMC12690471; doi:10.1093/icb/icaf026)
Supplement: icaf026_Supplemental_Files [file icaf026_supplemental_files.zip › icb-2025-0043-File010.docx]

**The effect of venipuncture site on hematology of bats: implications for comparative analyses: Supplemental Material**

Alicia Roistacher, Bret Demory, and Daniel J. Becker

**TABLE S1**

| **Cell Type** | **Blood** | **0.75% NaCl in mH20** | **0.5% Eosin Y** | **0.5% Crystal Violet** |
| --- | --- | --- | --- | --- |
| RBC | 1 | 1,175 | 24 | — |
| WBC | 6 | 582 | — | 12 |
| RET | 6 | 582 | — | 12 |

**TABLE S2**

| **Solution** | **95% EtOH** | **Glacial acetic acid** | **MeOH** | **mH20** | **Eosin Y** | **Crystal Violet** |
| --- | --- | --- | --- | --- | --- | --- |
| 0.5% EosinY, 0.1% glacial acetic acid, 20% v/v EtOH | 2 mL | 10 μL | — | 8 mL | 0.05 g | — |
| 0.5% Crystal violet, 20% v/v MeOH | — | — | 2 mL | 8 mL | — | 0.05 g |

**TABLE S3**

| **Cell Type** | **Variable** | **χ^2^** | **df** | ***p*** |
| --- | --- | --- | --- | --- |
| Monocyte | Vein | 0.0687 | 1 | 0.79324 |
|  | Species | 3.4638 | 1 | 0.06272 |
|  | Vein:Species | 0.5042 | 1 | 0.47766 |
| Basophil | Vein | 1.0455 | 1 | 0.30654 |
|  | **Species** | **5.1199** | **1** | **0.02365*** |
|  | Vein:Species | 0.1186 | 1 | 0.73055 |
| Segmented neutrophil | Vein | 1.3869 | 1 | 0.238937 |
|  | **Species** | **8.0534** | **1** | **0.004542**** |
|  | Vein:Species | 1.3914 | 1 | 0.238164 |
| Banded neutrophil | Vein | 0.1448 | 1 | 0.7035 |
|  | Species | 1.5605 | 1 | 0.2116 |
|  | Vein:Species | 0.8798 | 1 | 0.3483 |
| NL ratio | Vein | 0.4100 | 1 | 0.5219486 |
|  | **Species** | **12.0194** | **1** | **0.0005265***** |
|  | Vein:Species | 0.5411 | 1 | 0.4619589 |

**TABLE S4**

| **Cell Type** | **Variable** | **χ^2^** | **df** | ***p*** |
| --- | --- | --- | --- | --- |
| RBC | Vein | 0.8953 | 1 | 0.3440 |
|  | Species | 1.1201 | 1 | 0.2899 |
|  | Vein:Species | 2.5823 | 1 | 0.1081 |
| WBC | Vein | 0.9244 | 1 | 0.3363 |
|  | **Species** | **4.6806** | **1** | **0.0305*** |
|  | Vein:Species | 0.3688 | 1 | 0.5437 |
| RET | Vein | 1.6569 | 1 | 0.198028 |
|  | **Species** | **7.6330** | **1** | **0.005731**** |
|  | Vein:Species | 0.0008 | 1 | 0.976954 |
| Neutrophils | Vein | 1.2152 | 1 | 0.2703025 |
|  | **Species** | **14.4852** | **1** | **0.0001413***** |
|  | Vein:Species | 2.3206 | 1 | 0.1276740 |
| Lymphocytes | Vein | 0.1556 | 1 | 0.6932 |
|  | **Species** | **17.0460** | **1** | **3.649x10^-5^***** |
|  | Vein:Species | 101438 | 1 | 0.2849 |
| Monocyte | Vein | 0.4396 | 1 | 0.50731 |
|  | Species | 2.7862 | 1 | 0.09508 |
|  | Vein:Species | 0.5640 | 1 | 0.45266 |
| Basophil | Vein | 0.7313 | 1 | 0.3925 |
|  | Species | 2.1559 | 1 | 0.1420 |
|  | Vein:Species | 0.0272 | 1 | 0.8690 |
| Eosinophils | Vein | 0.0538 | 1 | 0.81665 |
|  | Species | 3.1564 | 1 | 0.07563 |
|  | Vein:Species | 0.0258 | 1 | 0.87236 |
| Banded neutrophil | Vein | 0.1796 | 1 | 0.6717 |
|  | Species | 1.7425 | 1 | 0.1868 |
|  | Vein:Species | 0.6061 | 1 | 0.4362 |
| Segmented neutrophil | Vein | 0.8802 | 1 | 0.3482 |
|  | **Species** | **16.0933** | **1** | **6.03x10^-5^***** |
|  | Vein:Species | 1.9286 | 1 | 0.1649 |
| NL ratio | Vein | 0.0191 | 1 | 0.890030 |
|  | **Species** | **10.8103** | **1** | **0.001009**** |
|  | Vein:Species | 0.4407 | 1 | 0.506797 |

**TABLE S5**

| **Cell Type** | **Variable** | **χ^2^** | **df** | ***p*** |
| --- | --- | --- | --- | --- |
| Neutrophils | Vein | 0.8724 | 1 | 0.350284 |
|  | **Species** | **6.8137** | **1** | **0.009046**** |
|  | Slide Size | 1.0382 | 1 | 0.308244 |
|  | Vein:Species | 1.7300 | 1 | 0.188417 |
| Lymphocytes | Vein | 0.6493 | 1 | 0.420370 |
|  | **Species** | **9.4046** | **1** | **0.002164**** |
|  | Slide Size | 0.0066 | 1 | 0.935230 |
|  | Vein:Species | 1.1965 | 1 | 0.274014 |
| Monocyte | Vein | 0.0555 | 1 | 0.81376 |
|  | Species | 3.4692 | 1 | 0.06252 |
|  | Slide Size | 0.0005 | 1 | 0.98218 |
|  | Vein:Species | 0.4974 | 1 | 0.48065 |
| Basophil | Vein | 1.2892 | 1 | 0.25619 |
|  | **Species** | **5.1413** | **1** | **0.02336*** |
|  | Slide Size | 0.6048 | 1 | 0.43675 |
|  | Vein:Species | 0.1047 | 1 | 0.74628 |
| Eosinophils | Vein | 0.1686 | 1 | 0.68134 |
|  | **Species** | **5.3966** | **1** | **0.02018*** |
|  | Slide Size | 3.0050 | 1 | 0.08301 |
|  | Vein:Species | 0.4068 | 1 | 0.52359 |
| Banded neutrophil | Vein | 0.1563 | 1 | 0.6926 |
|  | Species | 1.5623 | 1 | 0.2113 |
|  | Slide Size | 0.0360 | 1 | 0.8496 |
|  | Vein:Species | 0.8860 | 1 | 0.3466 |
| Segmented neutrophil | Vein | 0.5676 | 1 | 0.45123 |
|  | **Species** | **7.8542** | **1** | **0.00507**** |
|  | Slide Size | 1.0487 | 1 | 0.30580 |
|  | Vein:Species | 1.3075 | 1 | 0.25285 |
| NL ratio | Vein | 0.0791 | 1 | 0.778515 |
|  | **Species** | **7.8646** | **1** | **0.005041**** |
|  | Slide Size | 0.0322 | 1 | 0.857580 |
|  | Vein:Species | 0.7785 | 1 | 0.377608 |

**TABLE S6**

| **Cell Type** | **Variable** | **χ^2^** | **df** | ***p*** |
| --- | --- | --- | --- | --- |
| Neutrophils | Vein | 1.8670 | 1 | 0.17182 |
|  | **Species** | **5.8541** | **1** | **0.01554*** |
|  | Bleeding order | 0.7882 | 1 | 0.37464 |
|  | Vein:Species | 1.8013 | 1 | 0.17956 |
| Lymphocytes | Vein | 0.8387 | 1 | 0.35977 |
|  | **Species** | **7.3040** | **1** | **0.00688**** |
|  | **Bleeding order** | **4.1024** | **1** | **0.04282*** |
|  | Vein:Species | 1.2146 | 1 | 0.27043 |
| Monocyte | Vein | 0.8908 | 1 | 0.345252 |
|  | **Species** | **7.3487** | **1** | **0.006711**** |
|  | **Bleeding order** | **9.8903** | **1** | **0.001662**** |
|  | Vein:Species | 0.5120 | 1 | 0.474293 |
| Basophil | Vein | 1.7149 | 1 | 0.19035 |
|  | **Species** | **6.3871** | **1** | **0.01150*** |
|  | Bleeding order | 3.2471 | 1 | 0.07155 |
|  | Vein:Species | 0.1864 | 1 | 0.66596 |
| Eosinophils | Vein | 0.0987 | 1 | 0.753370 |
|  | **Species** | **3.8549** | **1** | **0.049601*** |
|  | **Bleeding order** | **7.7626** | **1** | **0.005334**** |
|  | Vein:Species | 0.2912 | 1 | 0.589456 |
| Banded neutrophil | Vein | 0.1367 | 1 | 0.7116 |
|  | Species | 1.6916 | 1 | 0.1934 |
|  | Bleeding order | 0.1730 | 1 | 0.6775 |
|  | Vein:Species | 0.8792 | 1 | 0.3484 |
| Segmented neutrophil | Vein | 1.3977 | 1 | 0.237114 |
|  | **Species** | **6.7332** | **1** | **0.009464**** |
|  | Bleeding order | 0.8609 | 1 | 0.353479 |
|  | Vein:Species | 1.3771 | 1 | 0.240600 |
| NL ratio | Vein | 0.0858 | 1 | 0.76954 |
|  | **Species** | **6.8509** | **1** | **0.00886**** |
|  | Bleeding order | 0.5112 | 1 | 0.47464 |
|  | Vein:Species | 0.6443 | 1 | 0.42217 |

**TABLE S7**

| **Cell Type** | **Variable** | **χ^2^** | **df** | ***p*** |
| --- | --- | --- | --- | --- |
| RBC | Vein | 0.7374 | 1 | 0.3905 |
|  | Species | 0.0000 | 1 | 0.9976 |
|  | Holding time | 0.0727 | 1 | 0.7875 |
|  | Vein:Species | 1.7814 | 1 | 0.1820 |
| WBC | Vein | 1.2387 | 1 | 0.26573 |
|  | Species | 1.6937 | 1 | 0.19311 |
|  | Holding time | 2.8293 | 1 | 0.09256 |
|  | Vein:Species | 0.5079 | 1 | 0.47604 |
| RET | Vein | 3.3149 | 1 | 0.068653 |
|  | **Species** | **10.8075** | **1** | **0.001011**** |
|  | Holding time | 3.0139 | 1 | 0.082554 |
|  | Vein:Species | 0.0075 | 1 | 0.931082 |
| Neutrophils | Vein | 1.4294 | 1 | 0.2319 |
|  | **Species** | **16.8495** | **1** | **4.046x10^-5^***** |
|  | Holding time | 2.4150 | 1 | 0.1202 |
|  | Vein:Species | 2.3059 | 1 | 0.1289 |
| Lymphocytes | Vein | 0.4825 | 1 | 0.4873 |
|  | **Species** | **19.6336** | **1** | **9.381x10^-6^***** |
|  | Holding time | 0.8977 | 1 | 0.3434 |
|  | Vein:Species | 1.6873 | 1 | 0.1940 |
| Monocyte | Vein | 0.1375 | 1 | 0.7108 |
|  | Species | 1.3700 | 1 | 0.2418 |
|  | Holding time | 0.0195 | 1 | 0.8890 |
|  | Vein:Species | 0.3366 | 1 | 0.5618 |
| Basophil | Vein | 0.4972 | 1 | 0.480716 |
|  | **Species** | **3.9032** | **1** | **0.048195*** |
|  | **Holding time** | **6.7332** | **1** | **0.009464**** |
|  | Vein:Species | 0.0275 | 1 | 0.868367 |
| Eosinophils | Vein | 0.3177 | 1 | 0.57299 |
|  | **Species** | **4.4684** | **1** | **0.03453*** |
|  | **Holding time** | **6.4316** | **1** | **0.01121*** |
|  | Vein:Species | 0.7230 | 1 | 0.39515 |
| Banded neutrophil | Vein | 0.5226 | 1 | 0.4697 |
|  | Species | 1.0333 | 1 | 0.3094 |
|  | Holding time | 0.6563 | 1 | 0.4179 |
|  | Vein:Species | 0.7896 | 1 | 0.3742 |
| Segmented neutrophil | Vein | 0.9708 | 1 | 0.3245 |
|  | **Species** | **18.4479** | **1** | **1.746x10^-5^***** |
|  | Holding time | 2.4115 | 1 | 0.1204 |
|  | Vein:Species | 1.8381 | 1 | 0.1752 |
| NL ratio | Vein | 0.0469 | 1 | 0.8284935 |
|  | **Species** | **12.5730** | **1** | **0.0003914***** |
|  | Holding time | 0.9022 | 1 | 0.3421922 |
|  | Vein:Species | 0.6069 | 1 | 0.4359683 |

**TABLE S8**

| **Cell Type** | **Variable** | **χ^2^** | **df** | ***p*** |
| --- | --- | --- | --- | --- |
| RBC | Vein | 0.1280 | 1 | 0.72048 |
|  | Species | 0.0072 | 1 | 0.93231 |
|  | Sex | 1.7048 | 1 | 0.19166 |
|  | Vein:Species | 3.3639 | 1 | 0.06664 |
| WBC | Vein | 0.8707 | 1 | 0.3507488 |
|  | **Species** | **5.1202** | **1** | **0.0236487*** |
|  | **Sex** | **11.3659** | **1** | **0.0007481***** |
|  | Vein:Species | 0.3557 | 1 | 0.5509178 |
| RET | Vein | 0.0010 | 1 | 0.97433 |
|  | **Species** | **5.6456** | **1** | **0.01750*** |
|  | Sex | 2.7840 | 1 | 0.09521 |
|  | Vein:Species | 0.0347 | 1 | 0.85229 |
| Neutrophils | Vein | 1.8028 | 1 | 0.179369 |
|  | **Species** | **7.5832** | **1** | **0.005891**** |
|  | Sex | 1.1348 | 1 | 0.286750 |
|  | Vein:Species | 1.7754 | 1 | 0.182710 |
| Lymphocytes | Vein | 0.8045 | 1 | 0.369759 |
|  | **Species** | **10.6551** | **1** | **0.001098**** |
|  | Sex | 2.7283 | 1 | 0.098581 |
|  | Vein:Species | 1.1833 | 1 | 0.276676 |
| Monocyte | Vein | 0.0946 | 1 | 0.758432 |
|  | Species | 2.6359 | 1 | 0.104472 |
|  | **Sex** | **8.3633** | **1** | **0.003829**** |
|  | Vein:Species | 0.5512 | 1 | 0.457842 |
| Basophil | Vein | 0.9893 | 1 | 0.31991 |
|  | **Species** | **5.0937** | **1** | **0.02401*** |
|  | Sex | 0.0000 | 1 | 0.99989 |
|  | Vein:Species | 0.1177 | 1 | 0.73154 |
| Eosinophils | Vein | 0.0110 | 1 | 0.91663 |
|  | **Species** | **5.4846** | **1** | **0.01918*** |
|  | Sex | 0.0084 | 1 | 0.92694 |
|  | Vein:Species | 0.2515 | 1 | 0.61599 |
| Banded neutrophil | Vein | 0.0950 | 1 | 0.7580 |
|  | Species | 1.2027 | 1 | 0.2728 |
|  | Sex | 1.4528 | 1 | 0.2281 |
|  | Vein:Species | 0.8864 | 1 | 0.3464 |
| Segmented neutrophil | Vein | 1.3363 | 1 | 0.247681 |
|  | **Species** | **8.5770** | **1** | **0.003404**** |
|  | Sex | 0.9364 | 1 | 0.333193 |
|  | Vein:Species | 1.3529 | 1 | 0.244772 |
| NL ratio | Vein | 0.0736 | 1 | 0.786207 |
|  | **Species** | **7.8088** | **1** | **0.005199**** |
|  | Sex | 0.9835 | 1 | 0.321335 |
|  | Vein:Species | 0.6450 | 1 | 0.421900 |
