# Supplementary material for: The Effect of Venipuncture Site on Hematology of Bats: Implications for Comparative Analyses
Source: Integr Comp Biol. 2025 May 19;65(6):1843–52. doi: 10.1093/icb/icaf026 (PMC12690471; doi:10.1093/icb/icaf026)
Supplement: icaf026_Supplemental_Files [file icaf026_supplemental_files.zip › icb-2025-0043-File011.docx]

|  |
| --- |
| **Figure 1.** Depiction of all venipuncture sites in bats. The externally visible veins are A) propatagial (*photo: Ralph Simon)*, B) brachial (*photo: Ralph Simon*), C) intrafemoral (*photo: Jon Alonzo*), D) lower intrafemoral, and E) upper brachial. The other veins that require lethal blood collection are shown with dashed lines. Here, we focus on hematological differences collected from the propatagial A) and intrafemoral C) veins. |

| **Figure 2:** Repeatability estimates (ICCs) and 95% confidence intervals for each cell type. Where confidence intervals are not present, values were exponentially negative. |
| --- |

**Figure 3.** Hematology values as a function of venipuncture site, stratified by bat species. Paired vein data for an individual bat are shown through line segments. Bold coloring indicates the predicted means and 95% confidence intervals for each vein per species from our GLMMs. Summary statistics are provided in Table 1 for effects of venipuncture site, bat species, and their interaction.

|  |
| --- |
